# A single layer spin-orbit torque nano-oscillator

**DOI:** 10.1038/s41467-019-10120-4

**Published:** 2019-05-29

**Authors:** Mohammad Haidar, Ahmad A. Awad, Mykola Dvornik, Roman Khymyn, Afshin Houshang, Johan Åkerman

**Affiliations:** 1 Physics Department, University of Gothenburg, 412 96 Gothenburg, Sweden; 2 Physics Department, Chalmers University of Technology, 412 96 Gothenburg, Sweden; 3 Material Physics and Nano Physics, School of Engineering Sciences, KTH Royal Institute of Technology, Electrum 229, 164 40 Kista, Sweden

**Keywords:** Magnetic properties and materials, Spintronics

## Abstract

Spin torque and spin Hall effect nano-oscillators generate high intensity spin wave auto-oscillations on the nanoscale enabling novel microwave applications in spintronics, magnonics, and neuromorphic computing. For their operation, these devices require externally generated spin currents either from an additional ferromagnetic layer or a material with a high spin Hall angle. Here we demonstrate highly coherent field and current tunable microwave signals from nano-constrictions in single 15–20 nm thick permalloy layers with oxide interfaces. Using a combination of spin torque ferromagnetic resonance measurements, scanning micro-Brillouin light scattering microscopy, and micromagnetic simulations, we identify the auto-oscillations as emanating from a localized edge mode of the nano-constriction driven by spin-orbit torques. Our results pave the way for greatly simplified designs of auto-oscillating nano-magnetic systems only requiring single ferromagnetic layers with oxide interfaces.

## Abstract (web summary)

Spin torque nano-oscillatiors promise novel microwave applications but the functioning relies on the spin current from additional ferromagnetic or metal layers. The authors here achieved in a single ferromagnetic layer sandwiched by nonmagnetic insulators the spin wave auto-oscillations due to a localized edge mode of the nano-constriction.

## Introduction

Spin transfer torque^1,2^ (STT)—the transfer of angular momentum from a spin current to the local spins—can act as negative spin wave damping and drive the magnetization of a ferromagnet (FM) into a state of sustained large-angle precession. For STT to occur, one first needs to generate a spin current and then inject this spin current into a ferromagnetic layer with a magnetization direction that is non-collinear with the polarization axis of the spin current. In so-called spin torque nano-oscillators (STNOs) these conditions can be fulfilled either in giant magnetoresistance trilayers, or in magnetic tunnel junctions, if the two ferromagnetic layers are in a non-collinear state^3^. More recently, the spin Hall effect^4–6^ was instead used to create pure spin currents injected into an adjacent ferromagnetic layer, in devices known as spin Hall nano-oscillators (SHNOs)^3^. These are both much easier to fabricate^7,8^, and exhibit superior synchronization properties and therefore much higher signal coherence^9–12^. Even so, SHNOs still suffer from a number of drawbacks and conflicting requirements: (i) the non-magnetic layer generating the spin current dramatically increases the zero-current spin wave damping of the ferromagnetic layer (up to 3× for NiFe/Pt^13^), (ii) the surface nature of the spin Hall effect requires ultrathin ferromagnetic layers for reasonable threshold currents, which further increases the spin wave damping, and (iii) since the current is shared between the driving layer and the magnetoresistive ferromagnetic layer, neither driving nor signal generation benefits from the total current, leading to non-optimal threshold currents, low output power, unnecessary dissipation, and heating. It would, therefore, be highly advantageous if one could do away with the additional metal layers for operation.

Spin-orbit torque can emerge due to a broken inversion symmetry^14–16^, either in the bulk of the magnetic material or at its interfaces. In particular, interfaces to insulating oxides^17–21^ can lead to a non-uniform spin distribution over the film thickness^22–24^. As oxide spin-orbitronics has seen considerable development in recent years^25,26^, oxide-based spin-orbit torque nano-oscillators with a single metallic layer could be envisioned. If so, the current would be confined to the ferromagnetic metal layer, avoiding the detrimental current sharing between adjacent metal layers and leading to an overall lower power consumption.

Here we propose, and experimentally demonstrate, a spin-orbit torque (SOT) nano-oscillator based on a single permalloy layer with thickness in the 15–20 nm range, grown on an Al_2_O_3_ substrate and capped with SiO_2_. The magnetodynamics of the unpatterned stacks is characterized using ferromagnetic resonance (FMR) spectroscopy and the linear spin wave modes of the final devices are determined using spin-torque FMR. Spin wave auto-oscillations are observed both electrically, via a microwave voltage from the anisotropic magnetoresistance, and optically, using micro-Brillouin Light Scattering (μ-BLS) microscopy. The current-field symmetry of the observed auto-oscillations agree with the expected symmetry for spin-orbit torques, either intrinsic to the Py layer itself or originating from the Al_2_O_3_/Py and Py/SiO_2_ interfaces. By performing additional thickness dependent spin-torque FMR measurements, we conclude that the anti-damping spin-torque originates from the interfaces in the devices.

## Results

### Characterization

**Fig. 1:**
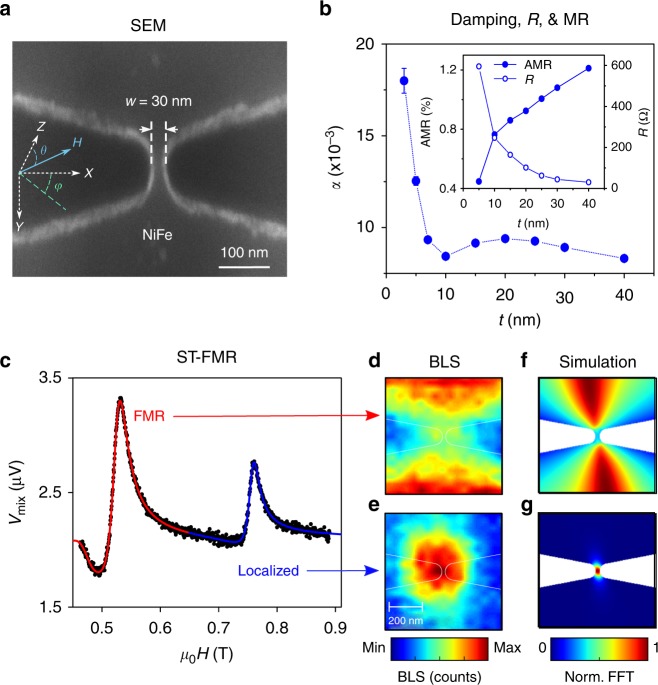
Nano-constriction fabrication, characterization, and simulation. **a** Scanning electron microscope image of a permalloy nano-constriction showing its width (*w* = 30 nm) and the direction of the applied field. **b** Gilbert damping of blanket permalloy films as a function of film thickness. Inset: AMR (closed symbol) and resistance (open symbol) vs. thickness of 30 nm wide nano-constrictions. **c** ST-FMR spectrum vs. field at *f* = 10 GHz for a *w* = 30 nm and *t* = 15 nm nano-constriction showing its two linear modes; red and blue lines are Lorentzian fits to the FMR mode and the localized mode, respectively. The spatial distribution of the FMR and the localized modes, imaged by μ-BLS (**d**, **e**), and calculated using micromagnetic simulations (**f**, **g**)

Figure 1a shows a scanning electron microscopy image of a *w* = 30 nm wide nano-constriction etched out of *t* = 15 nm thick single permalloy (Ni_80_Fe_20_; Py) layer, magnetron sputtered onto a sapphire substrate. A coplanar waveguide is connected to the nano-constriction for electrical measurements (not shown). During operation, an external magnetic field (*H*), applied at an out-of-plane angle *θ* = 15° and an in-plane angle *ϕ* = 22°, tilts the Py magnetization out of plane.

Figure 1b shows the thickness dependence of the Gilbert damping (*α*) of the unprocessed Py films as well as the resistance (R) and the anisotropic magnetoresistance (AMR) of the final devices (inset). As the thickness of the Py layer increases, we measure a noticeable decrease of *α* and R, and a corresponding increase of AMR^27^. Thus, using thicker permalloy films could be advantageous as compared to thinner films where by reducing *α* one can reduce the threshold current for driving spin wave auto-oscillations and by increasing AMR one can achieve larger output power as the electrical read-out is based on AMR in these devices.

### Magnetodynamics

To determine the magnetodynamic properties of the nano-constrictions, we first characterized them in the linear regime using spin-torque ferromagnetic resonance spectroscopy (ST-FMR)^28^ in a field applied at *θ* = 15° and *ϕ* = 22°. A typical ST-FMR spectrum as a function of field magnitude, and at constant frequency *f* = 10 GHz, reveals two well-separated resonances that can both be accurately fit with Lorentzians (Fig. 1c). The frequency vs. field dependence of the lower field peak agrees perfectly with the FMR dispersion at the specific angles used, from which we can extract an effective magnetization *μ*_0_*M*_eff_ = 0.72 T, by applying a Kittel-like formula. We can directly image the spatial distribution of the two linear modes using micro-Brillouin light scattering (μ-BLS) microscopy of the thermally excited SWs: the amplitude of the FMR mode is the largest well outside the nano-constriction region (Fig. 1d) whereas the amplitude of the higher-field (lower energy) mode is localized to the center of the nano-constriction (Fig. 1e). The localized mode is confined to the nano-constriction due to a local reduction of the internal magnetic field in this region. The nature of the observed modes is further corroborated by carrying out micromagnetic simulations where we calculate the spatial profile of the magnetization within the nano-constriction. The spatial maps for the simulated FMR mode and the localized mode (Fig. 1f, g) closely agree with the μ-BLS maps.

### Spin-torque driven magnetic oscillation

**Fig. 2:**
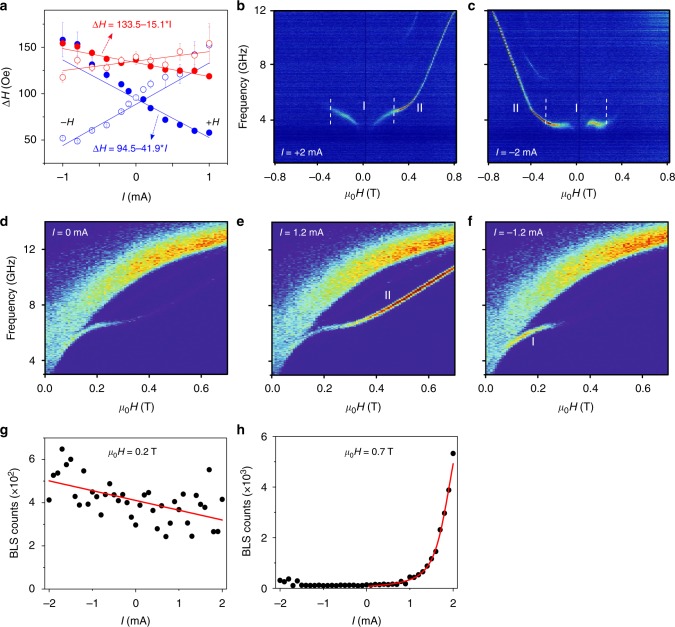
Current-induced spin wave auto-oscillations. **a** Current dependent ST-FMR linewidth of the FMR (red circles) and localized (blue circles) modes for positive field (filled symbols) and negative field (open symbols). The error bars are calculated using the standard error of Δ*H* while fitting the ST-FMR signals to a function consists of symmetric and anti-symmetric Lorentzian function. **b**, **c** Electrical measurements of the power spectral densities of spin wave auto-oscillations vs. applied field measured under a direct current of (**b**) +2 mA and (**c**) −2 mA. Label I and II indicate low-field and high-field auto-oscillations, respectively. **d**–**f**, μ-BLS measurements of the frequency vs. applied field showing the intensity of the FMR and the localized mode measured at 0 mA (**d**), +1.2 mA (**e**), and −1.2 mA (**f**). **g**, **h**, show the change of the BLS counts as a function of current at *μ*_0_*H* = 0.2 T and *μ*_0_*H* = 0.7 T. Solid red lines in **g**, **h**, are a linear fitting, and fitting to the theoretical prediction of Eq. (84a) in ref. ^29^, respectively

We then investigated the impact of a direct current on the linewidths (Δ*H*) of both the FMR and the localized modes (Fig. 2a). While the linewidths of both modes decreases with current magnitude for an even current-field symmetry (the orientation of the field and current satisfy **H** · **I** > 0) and increases for an odd symmetry (**H** · **I** < 0), the localized mode (blue circles) is three times more strongly affected than the FMR mode. This is again consistent with the localized mode residing in the nano-constriction region where the current density is the largest. Naively extrapolating the Δ*H* vs. current data to zero linewidth then predicts that auto-oscillations on this mode should be possible at a current magnitude of 2 mA.

That auto-oscillations are indeed possible to achieve at such currents is experimentally confirmed by sweeping the field magnitude in Fig. 2b, c. The electrical microwave power spectral density (PSD) is measured at constant currents of *I* = 2 mA (Fig. 2b) and *I* = −2 mA (Fig. 2c) as the field is swept from 0.8 to −0.8 T. Auto-oscillations are detected under positive applied fields between +0.1 and +0.8 T with a frequency tunable from 3.5 to 12 GHz. Somewhat unexpectedly, a measurable PSD is also observed under negative fields between −0.1 and −0.3 T where the frequency increases from 3.5 to 5.5 GHz. Then, by switching the direction of the current, (Fig. 2c), we measure a corresponding continuous branch of auto-oscillations under negative fields between −0.1 and −0.8 T and, again, a noticeable PSD appears under a positive field between +0.1 and +0.3 T, this time at an essentially constant frequency of about 4 GHz.

We categorize the measured PSD into two regions depending on the strength of the magnetic field: region (I) for low fields |*H*| ≤ 0.3 T and region (II) for high fields |*H*| > 0.3 T. In region (I) the PSD is independent of the field-current symmetry, i.e., a measurable PSD is always present regardless of the field and current polarities. The microwave signal in this region is rather weak with a large linewidth. In region (II) the auto-oscillations are both sharper and more powerful and are detected only when the orientation of the field and current satisfy **H** · **I** > 0. In this region, the spin current creates a spin-torque that is aligned either anti-parallel (**H** · **I** > 0) or parallel (**H** · **I** < 0) to the damping and hence it either suppresses or enhances the damping depending on symmetry. The spectral characteristics of the auto-oscillations are measured with a linewidth of 100 MHz and an integrated power of 2 pW.

### μ-BLS measurements

To better understand the origin of the two different regions and their distinctly different symmetries we performed μ-BLS measurements vs. field and current. Figure 2d shows the frequency vs. field of the thermally populated linear modes at *I* = 0 mA, where we observe the localized mode and the continuous SW band above the FMR frequency. When we increase the current to 1.2 mA (Fig. 2e) a noticeable increase in the intensity of the localized mode can be observed for high positive fields, i.e., in region (II). In contrast, by switching the current to −1.2 mA (Fig. 2f) the intensity of the localized mode in region (II) becomes weaker than at zero current, consistent with additional damping from spin-torque, while it increases slightly a low fields (region (I)). The BLS intensity demonstrates a rapid increase of the magnon population with the applied positive current (Fig. 2h) in the vicinity of the threshold value. The experimental data are in a good agreement with the theoretical prediction of Eq. (84a) in ref. ^29^ with the threshold current *I*_th_ = 1.6 mA, which supports our conclusion about spin-torque driven auto-oscillations. The current dependence in region (I) is however much weaker and essentially linear (Fig. 2g), suggesting thermal activation, but not auto-oscillations. We can hence conclude from the μ-BLS characterization that the observed auto-oscillations originate in a continuous manner from the localized mode inside the nano-constriction, similar to what has been demonstrated for the constriction SHNOs^30^, and that the auto-oscillations require a certain magnetic state reached only for a field above 0.3 T. These results were reproducible from device to device and auto-oscillations were observed for both *t* = 15 and 20 nm.

## Discussion

Next, we discuss the possible mechanism that could be driving the auto-oscillations, i.e., for which, the spin current should exert an opposite torque to the Gilbert damping with the observed current-field symmetry. Since our devices have a strongly non-uniform magnetization distribution in the vicinity of the constriction and should have a relatively strong temperature gradient during operation, we might have anti-damping originating from (i) Zhang-Li torque^31^, and (ii) spin torque from the spin-dependent Seebeck effect^32^. However, neither of these torques follows the experimentally observed **H** · **I** > 0 symmetry, as their sign would not depend on the direction of the applied current. The observed symmetry instead strongly suggests that spin-orbit torque (SOT) is the main source of anti-damping.

We identify the source of the angular momentum for the localized mode using μ-BLS. In the center of the constriction, we estimate the integrated amplitude of the auto-oscillating mode and the rest of the spin wave spectrum versus bias current. If the latter decreases with auto-oscillations amplitude, then the effect is intrinsic, analogous to Bose-Einstein condensation of magnons^33^. However, we observe that amplitudes of auto-oscillations and the rest of the magnon spectrum increase with the positive bias current, suggesting that angular momentum is pumped from the outside, either from the lattice, the interface, or both.

**Fig. 3:**
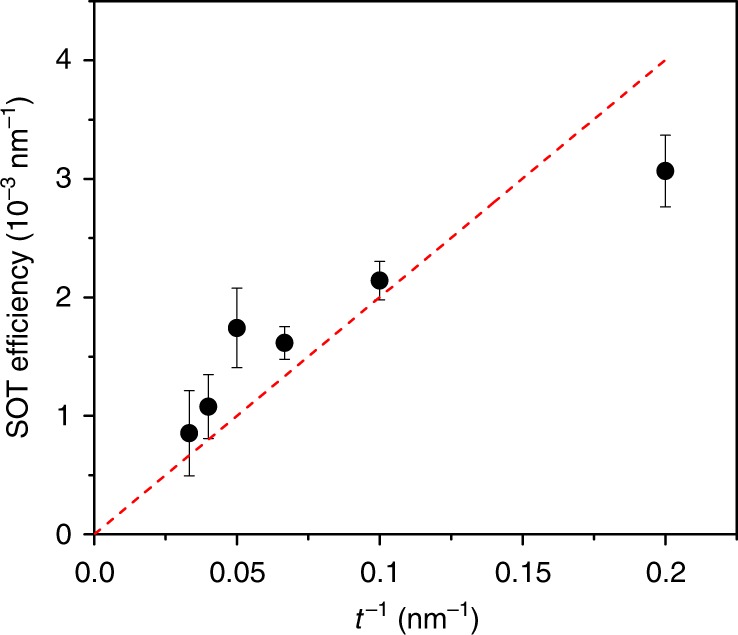
Spin-orbit torque efficiency. Thickness-dependent measurements of the in-plane spin-torque efficiency. Red dashed line shows a linear fit of the SOT efficiency to the inverse thickness. The error bars are calculated by Eq. (1) using the standard error of dΔ*H*/d*j*

To further determine whether the effect is of bulk or interfacial origin, we have measured the spin-torque efficiency, in devices of various thicknesses (*t* = 5–30 nm) using ST-FMR measurements with an in-plane magnetic field. We measured a common trend for all devices with different thicknesses similar to that presented in Fig. 2a: Δ*H* decreases (increases) linearly with positive (negative) current polarity under a positive applied field. We characterize the SOT efficiency as the ratio of the SOT angle and thickness of the device. Then, by using the approximation of the thin film, one can combine Eqs. (7) and (9) in^20^ and obtain:

1 $${\mathrm{SOT}}_{{\mathrm{eff}}} = \frac{{\mathrm{d}\Delta H}}{{\mathrm{d}j}}\frac{{eM_{\mathrm{s}}}}{{2\hbar \sin \phi }}\frac{{\omega ^2 + (\mu _0\gamma H_{\mathrm{r}})^2}}{{\mu _0\gamma \omega H_{\mathrm{r}}}},$$

where *ω* = 2*πf* is a resonant frequency and *H*_r_ is a resonant field. Our results, shown on Fig. 3, demonstrate the linear scaling of the SOT efficiency with the inverse thickness, which corroborates the interfacial origin of the spin torque in the devices.

Moreover, recent results obtained in ferromagnetic/oxide heterostructures suggest that SOT can result from non-equilibrium spin accumulation due to inversion symmetry breaking and enhanced spin-orbit coupling at the interfaces^14,34,35^. Although the dominant spin-orbit torque in our system is an interfacial effect, we cannot rule out minor contributions from other sources such as the intrinsic anomalous^36,37^ and spin Hall effects in Py^23,24^ and interfacial Rashba effect^17^. We note, however, that we can reproduce the experimental results, including the auto-oscillation threshold currents, using micromagnetic simulations, assuming a spin Hall-like SOT, and a spin Hall angle of about 0.13, which is in the range of values reported for Py/oxide stacks^26^.

Besides the fundamental interest of generating, controlling, and optimizing SOT in single ferromagnetic layers, e.g., using ultra-low damping metals and different oxide interfaces, our results will have a direct impact on a wide range of applications. A magnetic tunnel junction (MTJ) in the nano-constriction region only requires an additional ferromagnetic layer, which will allow for separate optimization of the SOT drive and the MTJ based high-power read-out. Arrays of SOT driven nano-constrictions can also be used for oscillator based neuromorphic computing^38^. In the sub-threshold regime, the intrinsic damping in magnonic crystals can be greatly reduced, solving their issues with high transmission losses^39^.

## Methods

### Sample fabrication

Single permalloy (Py, Ni_80_Fe_20_) layers with different thicknesses [*t* = 5–40 nm] were magnetron sputtered in a system with a base pressure lower than 3 × 10^−8^ Torr at room temperature onto a 18 mm × 18 mm pieces of sapphire C-plane substrate and in situ covered with 5 nm SiO_2_ to prevent the oxidation of the permalloy layer. The layers were then patterned into 4 μm × 12 μm rectangles with two 50 nm tip radii indentations forming nano-constrictions with nominal widths of 80–150 nm by e-beam lithography and subsequent argon ion milling using the negative e-beam resist as the etching mask. Ultra-small constriction widths were obtained by taking the advantage of ion beam milling at an angle of 45° with respect to the film normal and the associate lateral erosion of the etching mask. The constriction sizes were consistently reduced to 30 nm from their nominal values as measured by scanning electron microscopy. The devices were then covered with an additional 50 nm SiO2 layer to protect them from oxidation during measurements. A coplanar waveguide that provides electrical contact is connected to the nano-constriction by optical lithography followed by a lift off process of 980 nm of copper and 20 nm of gold.

### Ferromagnetic resonance measurements (FMR)

The magnetodynamic characteristics of the permalloy layers are determined by performing FMR measurements on unprocessed thin films using a NanOsc Instrument PhaseFMR system. Broadband FMR measurements were carried out with an in-plane field (*H*) and a coplanar waveguide where the FMR response is measured over the frequencies (*f*) range of 2–16 GHz. At each frequency, the resonance field (*H*_r_) and the FMR linewidth (Δ*H*) are extracted by fitting the FMR to a Lorentzian function. We determined the effective magnetization (*μ*_0_*M*_eff_) of thin films by fitting the dispersion relation to the Kittel equation. The Gilbert damping (*α*) is extracted by following the change of the FMR linewidth Δ*H* vs the frequency and using the relation $\Delta H = \frac{{2\alpha }}{\gamma }(2\pi f) + \Delta H_{\mathrm{0}}$, where Δ*H*_0_ is the inhomogeneous broadening. The results of *α* as a function of the film thickness are shown in (Fig. 1b). Note that Δ*H*_0_ is measured to be less than 0.2 mT in these films.

### Magnetoresistance of devices

In-plane angular dependent anisotropic magnetoresistance (AMR) measurement of the nano-constriction devices is performed using a rotatable projected field under a fixed external magnetic field of 0.1 T, with an applied dc current of 0.1 mA. We connect a Ground-Signal-Ground (GSG) pico-probe to the coplanar waveguide pads through which the devices are biased by a dc current. We measure the resistance while rotating the magnetic field in-plane. We use a Stoner-Wolhfarth model to fit the angular-dependence of AMR data where we assume a coherent rotation of the magnetization with the external field. The internal in-plane angle *ϕ*_int_ is then calculated using $\sin (\phi - \phi _{{\mathrm{int}}}) = \frac{{H_K}}{{2H}}\sin (2\phi _{{\mathrm{int}}})$ where *H*_*K*_ is a uniaxial anisotropy generated from the strong shape anisotropy in the vicinity of the nano-constriction associated with the current path.

### Spin torque-ferromagnetic resonance (ST-FMR) measurements

The magnetodynamics of the SOT nano-oscillator devices are studied by performing ST-FMR measurements. The devices are connected to a pulse-modulated signal generator to excite the dynamics and to a lock-in amplifier to detect the mixing voltage. The devices are placed in a uniform magnetic field that is oriented 15° out-of-plane, where the measurements were performed by sweeping the magnetic field at a fixed frequency, ranged between 4 and 14 GHz with an input power *P* = −20 dBm. The measured signals were fitted to a Lorentzain function having symmetric and anti-symmetric components to extract the resonance field and the linewidth. In the second step, we investigated the effect of the dc current on the magnetodynamics of the linear modes. We measure the ST-FMR spectra at 10 GHz while biasing the devices with a dc current between −1 and 1 mA. We follow the change of Δ*H* with the dc current under a positive and negative field directions for the FMR and localized modes as shown in (Fig. 1c).

### Microwave characterization

Microwave measurement were performed using a custom-built probe station where we mounted the sample with a fixed in-plane angle on a rotatable out-of-plane sample holder between the poles of an electromagnet. A direct electric current was applied to the devices through a high-frequency bias-T and the resulting rf oscillations amplified by a low-noise amplifier and recorded with a high-frequency spectrum analyzer (SA) using a low-resolution bandwidth of 1 MHz. All electrical microwave signals are from 16 averaged scans at each field, which reduces the scatter substantially. The auto-oscillations were detected electrically in tens of devices measured for each film thickness and the results were reproducible from device to device. The obtained spectra were then corrected to correspond to the power emitted by the device, taking into account the amplifier gain, the losses from the rf components and cables, and the impedance mismatch between the device and the 50 Ω measurement line. The spectra were fitted with a single symmetric Lorentzian to extract the auto-oscillation frequency, power, and linewidth.

### μ-BLS characterization

The magneto-optical measurements were performed using room temperature micro-focused BLS measurements. Spatially resolved maps of the magnetization dynamics are obtained by focusing a polarized monochromatic 532 nm single frequency laser (solid state diode-pumped) using a high numerical aperture (NA = 0.75) dark-field objective, which yields a diffraction limited resolution of 360 nm. The scattered light from the sample surface is then analysed by a high-contrast six-pass Tandem Fabry-Perot interferometer TFP-1 (JRS Scientific Instruments). The obtained BLS intensity is proportional to the square of the amplitude of the magnetization dynamics at the corresponding frequency.

### Micromagnetic simulations

The micromagnetic simulations were done using the GPU-accelerated finite-difference time-domain micromagnetic solver MuMax3^40^. The SOT nano-oscillator device is modeled on a 512 × 512 × 1 mesh with a cell size of 3.9063 × 3.9063 × 20 nm^3^ with a combination of preriodic and absorbing boundary conditions applied to mimic the extended geometry. The parameters used in the simulation include the saturation magnetization *μ*_0_*M*_S_ = 0.9 T, exchange stiffness *A* = 11 × 10^−12^ J m^−1^, the damping constant *α* = 0.009, and gyromagnetic ratio *γ* = 30 T**·**GHz^−1^ all determined from experiments on blanket films. In addition to the field *B* = 0.825 T applied 75° out-of-plane and 10° in-plane, we include the Oersted field landscape, calculated in COMSOL Multiphysics® simulation software.

In the simulations, the magnetization distribution is first relaxed to the ground state and, then, the linear modes are excited using a cardinal sine function with the amplitude of 10^−3^ T and the cut-off frequency of 30 GHz. The frequencies of the linear modes are obtained by applying Fast Fourier transform (FFT) on the simulated time traces of the averaged out-of-plane component of the magnetization(125 ns total with 50 GHz bandwidth). By performing FFT on each simulation cell using Semargl-ng software, full spatial maps of the generated FMR and localized modes are extracted.

## Data Availability

The data that support the findings of this study are available from the corresponding author upon reasonable request.
